# Climate Change Policy Coherence across Policies, Plans, and Strategies in Pakistan—Implications for the China–Pakistan Economic Corridor Plan

**DOI:** 10.1007/s00267-021-01449-y

**Published:** 2021-03-17

**Authors:** Abdul Waheed, Thomas Bernward Fischer, Muhammad Irfan Khan

**Affiliations:** 1 Department of Environmental Science, International Islamic University, Islamabad, Pakistan; 2 Environmental Assessment and Management Research Centre, School of Environmental Sciences, University of Liverpool, Liverpool, UK; 3 Research Unit for Environmental Science and Management, Faculty of Natural and Agricultural Sciences, North West University (Potchefstroom Campus), Potchefstroom, South Africa

**Keywords:** Climate change policy coherence, Adaptation and mitigation, CPEC, Pakistan

## Abstract

Climate Change (CC) adaptation and mitigation policy coherence (PC) across sectors is essential to effectively address CC challenges and support synergies. Pakistan is highly vulnerable to CC. In this paper, the extent to which Pakistan’s national and provincial water, agriculture, and energy sector policies, development plans and strategies are aligned in a CC policy coherent manner is established. In this context, a qualitative content document analysis with associated scoring is used to assess government documents. Furthermore, implications of the China–Pakistan Economic Corridor Initiative (CPEC; 2017–2030), the biggest infrastructure investment program ever in Pakistan, are discussed. An important result is that sectoral policies are found to have different degrees of PC. Better coherence is found at federal than at provincial levels. Furthermore, CC policies are found to be more coherently addressed in water and agriculture policies than in energy policies. It is suggested that to achieve higher levels of CC PC, federal and provincial governments should establish mechanisms of intergovernmental consultation for policy-making and cross-sectoral planning, especially in the energy sector. Our findings can help the Government of Pakistan to transform CPEC into a model green Belt and Road Initiative (BRI) in the region. In this context, there are important implications with regards to e.g., reducing coal-based energy projects and environmentally damaging infrastructure activities in sensitive ecosystems. With this paper, the authors want to raise awareness of the key importance of CC PC, particular in context of the BRI. Many countries participating in the initiative have carbon reduction targets in place.

## Introduction

Pakistan is highly vulnerable to climate change (CC) (Malik et al. 2012), which is threatening the country’s water, food, and energy security (GoP 2012b). The country has experienced numerous climate-related disasters in recent year (Mukhtar 2018), including floods, droughts, storms, avalanches, glacial lack outburst, and landslides (Ali and Kandhro 2015). This is said to have resulted in a damage of US$ 20B over the past two decades (Shaw 2015). In the 2010 floods alone, 20 million people and 1/5th of the country’s geographical area were directly affected (GoP 2012a). Whilst improved agronomical and conservation practices as well as more efficient resource use can help adapting to CC (Hellin et al. 2014; Howden et al. 2007), to what the extent these can be implemented in a developing country like Pakistan is uncertain. Although Pakistan is contributing to <1% of the world’s GHGs emissions (Mohydin 2019), it has been said to be the seventh most CC-affected country (Eckstein et al. 2018). In addition to socio-economic loss, Pakistan faces serious environmental problems and the cost of environmental degradation in 2015 was said to be 9% of GDP equivalent (up from 6% of GDP in 2006) (Bank 2006; GoP 2017b). However, the government is currently spending just 0.00028% of GDP on environmental protection, including CC mitigation and adaptation (Khan 2016).

The China–Pakistan Economic Corridor (CPEC) is the biggest investment project in Pakistan ever, worth US $62B. There are likely to be numerous environmental and CC impacts associated with CPEC investment (Zubedi et al. 2018; Kouser et al. 2020), in particular in connection with coal-based energy projects and infrastructure development.

Pakistan intends to reduce its expected GHG emissions by up to 20% of (equivalent to 1603 MtCO_2_) by 2030, subject to funding (GoP 2016; Hussain et al. 2019b). This amounts to US$ 40B at 2016 prices and climate adaptation costs are projected to be US$ 7–14B/annum (GoP 2016), while mitigation costs for Pakistan are ranging between US $8B and US $17B by 2050 (GoP and UNFCC 2011). The energy sector is the main contributor to GHG emissions (50%), followed by agriculture (39%), industrial processes (6%), and other activities (5%) (GoP 2010).

The agricultural sector of Pakistan contributes nearly 21% to its GDP and to over 43% to the livelihood of rural populations (GoP 2015a). Water availability has dropped from 5000 m^3^ in 1951 to current levels of <1100 m^3^ per person (GoP 2014). In the future, CC could significantly affect water availability and thus agriculture (Qureshi 2005; GoP 2012b).

CC adaptation and mitigation are cross-cutting issues and need to be dealt within an integrated manner (Birkmann and Von Teichman 2010), for example, through strategic environmental assessment (SEA) of policies, plans, and programs (Fischer 2007), and environmental impact assessment of projects (Jiricka-Purrer et al. 2018). To date, research has highlighted numerous CC planning challenges; for example, a lack of cross-sectoral (Rahman 2010) and coherent (Khan and Jan 2015) planning, as well as a lack of institutional coordination (Chaudhry 2017; Ahmed et al. 2020). CC policy coherence (PC) is an area, which has not yet been examined in Pakistan. In this context, there are particular concerns with regards to the CPEC.

The objectives of this paper are:

(i) to establish the extent to which CC adaptation and mitigation are mainstreamed into water, agriculture, and energy sector policies, development strategies and plans, and in disaster risk management plans in Pakistan;
(ii) to analyse coherence in these policies, strategies, and plans with regards to CC adaptation and mitigation; and
(iii) to critically review the CPEC development plan in the light of the above.

## Conceptual Framework

PC deals with compatibility across policies along the entire policy cycle, from policy objectives to impacts (Nilsson et al. 2012). It promotes synergies between and within different policies (Nilsson et al. 2012). Whilst there is no universally agreed definition of PC, it is said to lead to policy stability and reduced policy failure (Howlett and Rayner 2007). Policy incoherence causes coordination and implementation problems (Cohen et al. 2017) and leads to an inefficient usage of resources (Mallory 2016). Consistency within sector-specific policies and plans is a pre-condition of PC (Voyer et al. 2020). Internal PC refers to interactions between policy objectives within a single policy domain, and external PC means interaction of different policies (Nilsson et al. 2012). CC PC is about coherence between CC adaptation and mitigation within and across policy domains (Nilsson et al. 2012). Similarly, internal CC PC promotes synergies and co-benefits (Di Gregorio et al. 2017) and reduces negative interactions. External CC PC refers to mutually beneficial practices and trade-offs reduction between CC aims and non-CC objectives (Di Gregorio et al. 2017).

The concept of PC with regards to CC and sustainable development has been examined by various authors. For example, CC PC among water, energy, land, food, and climate policies was assessed by Papadopoulou et al. (2020). Kalaba et al. (2014) analyzed PC between CC, agriculture, forest, and energy polices for Zambia and (Scobie 2016) highlighted challenges for small island developing states. These were said to include inadequate political will, and lack of accountability among actors. Similarly, bureaucratic politics were found to undermine policy integration and CC PC in Indonesia (Di Gregorio et al. 2017). Ranabhat et al. (2018) suggested that a collaborative and multi-stakeholder approach was required for effective CC PC in Nepal. Benson and Lorenzoni (2017) studied PC and CC adaptation in flood risk management plans in the UK and (England et al. 2018) assessed CC adaptation and PC across sectoral polices in South Africa.

Pakistan is a signatory to the Sendai Framework for Disaster Risk Reduction 2015–2030 (UN 2015), the Paris Agreement on CC (UNFCC 2015), and the sustainable development goals (SDGs). All of those stress the importance of PC. With regards to the SDGs, all members have “to pursue PC and an enabling environment for sustainable development at all levels and by all actors” (UN 2016). Pakistan’s overarching National Climate Change Policy (NCCP) 2012 is committed to integrate CC and the environment into development policies (GoP 2012b). CC mainstreaming requires PC and the reduction of inconsistent policies, creating opportunities for synergies (Juhola and Westerhoff 2011).

Pakistan is facing challenges with regards to policy formation and execution that lead to a reduction of PC. First, stakeholder consultations have not been given due consideration in policy formation. Ghani (2014) suggested that consultation, deliberation, and debate among stakeholders are hindered by high decision-making bureaucrats in policy formation processes. Furthermore, the former governor of the State Bank of Pakistan stated that capacity of provincial ministries and departments was inadequate in preparing policy documents, due to lack of essential knowledge and competence (Husain 2013) and that “Inter-ministerial consultation is more hostile than cooperative in nature. Ministers feel personally offended if their policy documents are criticized by other ministers. Stakeholder consultation is superficial and views of stakeholders, if diverting from those preparing them, do not find any place in the revised documents” (Husain 2013, p.7).

Second, effective execution of a policy requires availability of adequate resources (Gerston 2010). In the case of Pakistan, due to financial constraints and capacity building issues, these resources are not utilized properly in policy development processes. Similarly, other factors explaining the lack of consistency include poor institutional coordination, corruption, and weak policy formulation and implementation processes (Sirajul 2015). Sectoral policies mention the adoption of integrated management (e.g., integrated water management) but fail to outline any implementation frameworks. Integrated water resources management cannot be executed due to national and provincial mandates on water resource management, and associated equitable dissemination of costs and benefits among stakeholders (Khan 2019). Finally, contradictory interests lead to policy incoherence (Siitonen 2016).

## CC and China–Pakistan Economic Corridor Plan (CPEC)

The CPEC is an ongoing development plan (2017–2030) worth US $62B under China’s Belt and Road Initiative (BRI) (Kouser et al. 2020). It is considered a geopolitical and economic game changer in the region. From CPEC, Pakistan will receive 70% of total investment as foreign direct investment (FDI) (Husain and Arrfat 2018). CPEC’s monetary value is greater than the accumulated FDI to Pakistan since 1970 (Ghani and Sharma 2018). The main investment will be in energy and infrastructure projects (Mehar 2017; Vats 2016) that are expected to accelerate GDP growth to 7.5% pa by 2030 (Mirza et al. 2019). The key cooperative areas of the CPEC plan (2017–2030) are connectivity (infrastructure development), energy, trade, industrial parks development, agriculture, tourism, and financial cooperation. It has four priorities, namely the Gwadar port, energy, infrastructure construction, and industrial cooperation (GoP 2017a).

**Fig. 1:**
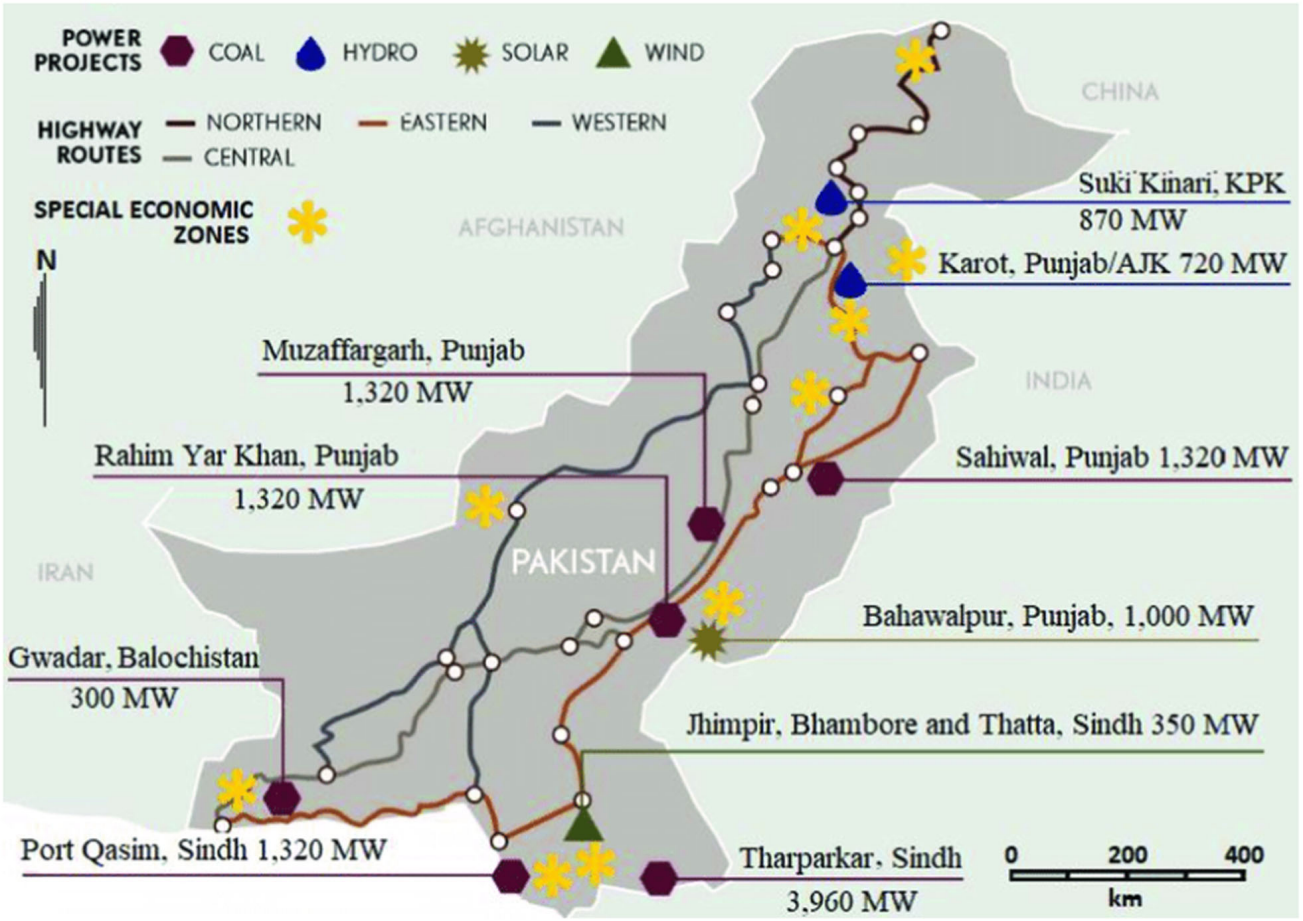
Majors CPEC projects (Farooqui and Aftab 2018)

There are obvious inconsistencies, including e.g., the CPEC coal power projects that are not consistent with Pakistan’s own green policies (Ebrahim 2020). The government’s alternative energy policy (2019) commits to 30% of energy mix being from renewable sources by 2030 (GoP 2019a, b). It is within this context that subsequently an analysis of CC mitigation and adaptation coherence across sectoral and CC policies, development strategies, and plans is provided. Figure 1 shows major CPEC projects planned in Pakistan.

CPEC projects are expected to result in an increase in GHG emission of 371 MtCO_2_ by the year 2030 (Janjua et al. 2018). The energy sector will make up around 56% of Pakistan’s total annual GHG emissions in 2030 (Janjua et al. 2018). Most energy projects of CPEC are coal-based with severe environmental and potentially social impacts (Bilgen 2016; Verma et al. 2017). Connected with CPEC, about 7000 trucks per day are expected to go through Northern Pakistan, resulting in emissions of carbon dioxide of up to 36.5 million tons (Qazilbash 2017).

The agriculture and energy sectors are key development areas of CPEC 2017–2030 (GoP 2017a) contributing significantly to GHG emissions. Energy sector emissions have increased by 117% since 2015 and those associated with agriculture have increased by 145% (GoP 2016). Water, energy, and food sectors are particularly vulnerable to CC (Hussain et al. 2019a). However, coordinated, cross-sectoral, and multiscale CC adaptation and mitigation planning has been missing (Rahman 2010).

## Methodology

Qualitative document analysis (Altheide et al. 2008) and content analysis (Steve 2001) are the basis for the empirical research into PC underlying this paper. A scoring system based on the work of Le Gouais and Wach (2013) was used for establishing sector rankings, followed by a validation based on semi-structured interviews with experts and practitioners. Stages of the project underlying this paper include (i) establishment of criteria of document selection, (ii) identification of relevant documents, (iii) document analysis, (iv) validation, and last (v) finalization (Altheide et al. 2008). At stage (i), official government documents were considered from relevant ministries and departments of Federal and provincial governments of Punjab (PB), Sindh (SD), Khyber Pakhtunkhwa (KP), Baluchistan (BA), Gilgit-Baltistan (GB) region and the territory of Azad Jammu and Kashmir (AJK). Whilst Federal and provincial governments of PB, KP and GB had complete sets of policy documents across water, agriculture, energy, and CC policies, strategies, development plans, and programs (Table 1), in SD, BA, and AJK, only few such documents exist. Therefore, these were not considered.

**Table 1:** Policy documents of selected provinces for qualitative document analysis

Sectoral policies/strategies/plans	Documents of Federal, Punjab, Gilgit-Baltistan, and Khyber Pakhtunkhwa governments			
	Federal	Punjab	Gilgit-Baltistan	Khyber Pakhtunkhwa
Water	National Water Policy (GoP 2018b)	Punjab Water Policy (GoPB 2018d)	Adopted National Water Policy (GoGB 2018a)	Water Policy 2015 (GoKP 2015)
Agriculture	National Food Security Policy (GoP 2018a)	Punjab Agriculture Policy (GoPB 2018b)	Provincial Agriculture Sector Policy (Draft) (GoGB 2018b)	Agriculture Policy (GoKP 2013)
Energy	National Power Policy (GoP 2013b)	Punjab Power Generation Policy (GoPB 2009)	Adopted Power Generation Policy (GoP 2015b)	Hydro Power Policy 2016 (GoKP 2016a, b)
Climate	National Climate Change Policy (GoP 2012b)	Punjab Climate Change Policy (Draft) (GoPB 2017)	Climate Change Strategy and Action Plan (GoGB 2017b)	Climate Change Policy (Draft) (GoKP 2016a, b)
Development plans and programs	Annual Plan 2019–2020 (GoP 2019a, b)	Development Program 2018–2019 (GoPB 2018a)	Annual Development Plan 2017–2018 (GoGB 2017a)	Annual Development Program 2018–2019 (GoKP 2018a)
Development strategy	National Sustainable Development Strategy (GoP 2017b)	Punjab Growth Strategy (GoPB 2019)	Strategy for Sustainable Development (GoP and IUCN 2003)	Sustainable Development Strategy (GoKP 2019b)
Disaster management	National Disaster Management Plan 2012–2022 (GoP 2012c)	Disaster Risk Management Plan 2008 (GoPB 2008)	Disaster Risk Management Plan Northern Area 2008 (GoGB 2008)	Monsoon Contingency Plan 2019 (GoKP 2019a)

The subsequent focus is on water, agriculture and energy policies, strategies and plans, disaster management plans, as well as on cross-sectoral linkages for mainstreaming CC. For collection of relevant policy documents, websites of federal ministries and provincial departments (stage ii) were used. In case, policy documents were not placed on websites, officials were asked (by phone and/or email) to provide them. Documents were then systematically analyzed (stage iii) with regards to: whether CC adaptation and mitigation were mentioned; how they were being addressed, i.e., whether they were mentioned as generic statements or in policy objectives, and/or with detailed plans, activities, implementation frameworks; and whether statements of CC adaptation and mitigation were consistent with other policy documents. A four-step content analysis approach was used for document analysis (Steve 2001). The PC assessment criteria are depicted in Table 2.

**Table 2:** Policy coherence assessment scoring criteria (Le Gouais and Wach 2013)

Coherence category	Coherence narrative	Score	Symbol
High coherence	The policy document aligns across water, agriculture, energy sectors, and statements for climate change. Policy documents offer attention to water–agriculture–energy inter-sector alignment to adapt to and mitigate CC, activities, strategies, plans, and implementation framework.	3	✓✓
Partial coherence	Though water–agriculture–energy inter-sector alignment are considered in policy documents to adapt and mitigate CC, mechanisms to achieve it are not well-defined. A few activities strategies, & implementation framework are incorporated but fail to incorporate comprehensive activities strategies, & implementation framework.	2	✓
Limited coherence	The policy document in general statements (i.e., no specific approaches or plans) supports water–agriculture–energy inter -sector alignment to adapt and mitigate CC. But no details are presented for activities, plans, and implementation frameworks.	1	⇔
No coherence	No evidence found that sectoral policy statements are harmonized and/or aligned.	0	✗

Selected policies, as well as development plans and programs were assessed with regards to the presence of five key subjects: (a) water, (b) agriculture, (c) energy, (d) water–agriculture–energy inter-sectoral alignment to adapt to and mitigate CC, and (e) CC adaptation and mitigation (see Table 3). When CC adaptation and mitigation were included was taken note of and the context in which they were found in was described. Words used in the analysis of each selected policy document include:

- Flood and drought management,
- Disaster risk reduction (DRR),
- Disaster management,
- Water security,
- Food and agriculture security,
- Environmental protection,
- Energy security,
- CC adaptation and mitigation,
- PC/coordination,
- Integrated planning/management.

**Table 3:** Coherence in policy documents with respect to five key subjects and adaptation and mitigation keywords for Federal, Punjab, Gilgit-Baltistan, and Khyber Pakhtunkhwa governments

Key subjects	WP	AP	EP	CCP	DP	DS	DMP
Federal							
Water	N/A	✓	⇔	✓✓	✓	✓✓	✓
Agriculture	✓	N/A	✗	✓✓	✓	✓✓	✓
Energy	✓	✓	N/A	✓	✓	✓✓	✓
Water–agriculture–energy inter-sectors alignment for climate change adaptation and mitigation	✓	✓	⇔	✓✓	✓	✓✓	✓✓
Climate change adaptation and mitigation	✓	✓	⇔	✓	✓	✓✓	✓
Total	8	8	3	13	10	15	11
Mean	2	2	0.75	2.6	2	3	2.2
%	●	●	◉	□	●	■	●
Punjab							
Water	N/A	✓	⇔	✓	✓	✓	✓
Agriculture	⇔	N/A	✗	✓	✓	✓	✓
Energy	✗	✓	N/A	✓	✓	✓	✗
Water–agriculture–energy inter-sectors alignment for climate change adaptation and mitigation	⇔	✓	⇔	✓	✓	✓	⇔
Climate change adaptation and mitigation	✓	✓	⇔	✓	✓	✓	⇔
Total	4	8	3	10	10	10	6
Mean	1	2	0.75	2	2	2	1.2
%	◉	●	◉	●	●	●	◉
Khyber Pakhtunkhwa							
Water	N/A	✓	⇔	✓.	✓	✓	⇔
Agriculture	✗	N/A	✗	✓	⇔	✓	⇔
Energy	✗	⇔	N/A	✓	⇔	⇔	✗
Water–agriculture–energy inter-sectors alignment for climate change adaptation and mitigation	⇔		⇔	✓	✓	✓	⇔
Climate change adaptation and mitigation	⇔	✓	⇔	✓	✓	✓	⇔
Total	2	5	3	10	8	9	4
Mean	0.5	1.25	0.75	2	1.6	1.8	0.8
%	○	◉	◉	●	●	●	◉
Gilgit-Baltistan							
Water	N/A	✓	⇔	✓	⇔	⇔	✓
Agriculture	✓	N/A	✗	✓	⇔	⇔	✓
Energy	✓.	⇔	N/A	⇔	⇔	⇔	⇔
Water–agriculture–energy inter-sectors alignment for climate change adaptation and mitigation	✓	✓	⇔	✓	⇔	⇔.	⇔
Climate change adaptation and mitigation	✓	✓	⇔	✓	⇔	✗	✓
Total	8	7	3	9	5	4	8
Mean	2	1.75	0.75	1.8	1	0.8	1.6
%	●	●	◉	●	◉	◉	●

✓✓ = 3 indicates high coherence i.e., the policy document aligns across water, agriculture, energy sectors, and statements for climate change. Policy documents offer attention to water–agriculture–energy inter-sector alignment to adapt to and mitigate CC, activities, strategies, plans, and implementation framework; ✓ = 2 indicate partial coherencies i.e., though water–agriculture–energy inter-sector alignment are considered in policy documents to adapt and mitigate CC, mechanisms to achieve it are not well-defined. A few activities strategies, & implementation framework are incorporated but fail to incorporate comprehensive activities strategies, & implementation framework; ⇔ = 1 indicates limited coherence i.e., policy document in general statements (i.e. no specific approaches or plans) support water–agriculture–energy inter-sector alignment to adapt and mitigate CC. But no details are provided about activities, plans, and implementation framework; × = 0 no coherence i.e., no evidence found that sectoral policy statements are harmonized and/or aligned; ■ = 100%; □ = 75–99%; ● = 50–74%; ◉ = 25–49%; ○ = <25%

*WP* water policy, *AP* agriculture policy, *EP* energy policy, *DS* development strategy, *CCP* climate change policy, *DP* development plan, *DMP* disaster management plan

Analysis was conducted for documents from each of the selected provinces and territories, making cross-comparisons of sectors, development plans, strategies, and policies possible. Policy development dates were recorded. Furthermore, information obtained was used to guide expert interviews. Allocated scores range from 3 (full coherence) to 0 (no coherence). By calculating the average of two values (mean of means), we assessed the coherence of policies relative to one another within each province/territory (Table 4). For example, the coherence of the Federal’s Water Policy (2018) in relation to its CC Policy (2012) is 2.3 (with an average coherence being 2 for Federal’s Water Policy and 2.6 for its CC Policy). This average value meant that there was a partial PC score. Validation and finalization involved semi-structured interviews with experts that work across the various sectors. For this purpose, assessment results of Tables 3 and 4 were discussed with experts. To ensure confidentiality, no information is provided on the role of or relationship with interviewees. Interview records were coded according to sectoral themes and policy priority areas. Finally, the CPEC development plan was assessed in the light of the results on PC in other decision processes of Pakistan, based on the criteria shown in Table 2.

**Table 4:** Coherence score across sectoral policies, strategies, and plans at federal and provincial level

Sectoral policies/strategies/plans		Water policy	Agriculture policy	Energy policy	Climate change policy	Development plan	Development strategy	Disaster management plan	Total coherence score	Maximum possible coherence score
Federal	National Water Policy 2018 (GoP)		2	1.37	2.3	2	2.5	2.1	12.27	18
	National Food Security Policy 2018 (GoP)	2		1.37	2.3	2	2.5	2.1	12.27	18
	Energy Policy 2013 (GoP)	1.37	1.37		1.76	1.37	1.87	1.47	9.21	18
	National Climate Change Policy 2012 (GoP)	2.3	2.3	1.76		2.3	2.8	2.4	13.86	18
	Annual Developmental Plan 2018–2019 (GoP)	2	2	1.37	2.3		2.5	2.1	12.27	18
	National Sustainable Development Strategy 2017 (GoP)	2.5	2.5	1.87	2.8	2.5		2.6	14.77	18
	Disaster Management Plan 2012–2022 (GoP)	2.1	2.1	1.47	2.4	2.1	2.6		12.77	18
	Total coherence score	12.27	12.27	9.21	13.86	12.27	14.77	12.77	87.32	126
	%	●	●	●	□	●	□	●	●	■
Punjab	Water Policy 2018 (GoPB)		1.5	0.87	1.5	1.5	1.5	1.1	7.97	18
	Punjab Agriculture Policy 2017(GoPB)	1.5		1.37	2	2	2	1.6	10.47	18
	Power Generation Policy 2006 revised in 2009 (GoPB)	0.87	1.37		1.37	1.37	1.37	0.97	7.32	18
	Climate Change Policy 2017(Draft) (GoPB)	1.5	2	1.37		2	2	1.6	10.47	18
	Development Program 2018–19 (GoPB)	1.5	2	1.37	2		2	1.6	10.47	18
	Punjab Growth Strategy 2023 (GoPB)	1.5	2	1.37	2	2		1.6	10.47	18
	Disaster Risk Management Plan 2008 (GoPB)	1.1	1.6	0.97	1.6	1.6	1.6		8.47	18
	Total coherence score	7.97	10.47	7.32	10.47	10.47	10.47	8.47	65.64	126
	%	◉	●	◉	●	●	●	◉	●	■
Khyber Pakhtunkhwa	Water Policy 2015 (GoKP)		0.87	0.62	1.25	1.05	1.15	0.65	5.59	18
	Agriculture Policy (2013–2023) (GoKP)	0.87		1	1.62	1.42	1.52	1.02	7.45	18
	Hydro Power Policy 2016 (GoKP)	0.62	1		1.37	1.17	1.27	0.77	6.2	18
	Climate Change Policy 2016 (GoKP)	1.25	1.62	1.37		1.8	1.9	1.4	9.34	18
	Annual Development Program 2019–2020 (GoKP)	1.05	1.42	1.17	1.8		1.7	1.2	8.34	18
	Sustainable Development Strategy 2019–2023 (GoKP)	1.15	1.52	1.27	1.9	1.7		1.3	8.84	18
	Monsoon Contingency Plan 2019 (GoKP)	0.65	1.02	0.77	1.4	1.2	1.3		6.34	18
	Total coherence score	5.59	7.45	6.2	9.34	8.34	8.84	6.34	52.1	126
	%	◉	◉	◉	●	◉	◉	◉	◉	■
Gilgit-Baltistan	National Water Policy 2018 (GoP) Adopted		1.87	1.37	1.9	1.5	1.4	1.8	9.84	18
	Agriculture Sector Policy 2018 (GoGB)	1.87		1.25	1.77	1.37	1.27	1.67	9.2	18
	Power Generation Policy 2015 (GoGB)	1.37	1.25		1.27	0.87	0.77	1.17	6.7	18
	Climate Change Strategy and Action Plan 2017 (GoGB)	1.9	1.77	1.27		1.4	1.3	1.7	9.34	18
	Annual Development Plan 2017–2018 (GoGB)	1.5	1.37	0.87	1.4		0.9	1.3	7.34	18
	Strategy for Sustainable Development 2003 (GoGB)	1.4	1.27	0.77	1.3	0.9		1.2	6.84	18
	Disaster Risk Management Plan Northern Area 2008	1.8	1.67	1.17	1.7	1.3	1.2		8.84	18
	Total coherence score	9.84	9.2	6.7	9.34	7.34	6.84	8.84	58.158.1	126
	%	●	●	◉	●	◉	◉	◉	◉	■

■ = 100%, □ = 75–99%, ● = 50–74%, ◉ = 25–49%, ○ = <25%. Coherence score of policies with one another and within each province/territory calculated by taking average of two values (mean of means) from Table 3.

## Results and Discussion

### CC Adaptation and Mitigation Mainstreaming in Sectoral Policies, Development Plans, Strategies and Disaster Management Plans

#### Water Sector Policies

National Water Policy aims at restoring and maintaining the health of the environment and water-related ecosystems. Associated planning principles include that environmental sustainability must be ensured, and EIA studies be carried out concurrently with project feasibility studies for water resources. Consistency with economic viability, social acceptability, and environmental sustainability also needs to be ensured. CC mitigation and adaptation assessment should be carried out “for sustainable water resource development and management” to address water, energy and food security, and climate-driven disasters. Although water is a national responsibility, agriculture and irrigation, environment and water-related sub-sectors are provincial subjects under the 18th constitutional amendment (GoP 2018b). KP and GB have still not developed their own policies for water management, with GB having adopted the National Water Policy, and KP having formulated a drinking Water Policy in 2015. This highlights the need for water resource conservation, and commits to “Measures [that] will be taken to identify, protect, develop, and conserve surface and ground water resources in line with Provisions of National Environmental Policy (NEP) 2005 and KP Environmental Act 2014.” It is also stated that “Due consideration will be given to the adverse impacts of CC, vulnerability and fragility, in planning and development of water supply scheme” (GoKP 2015). However, adaptation and mitigation measures for water resource management are not mentioned.

PB’s Water Policy states that “policy measures related to water resources applicable to PB in line with the NCCP 2012 [should] be adopted” but clear delivery mechanisms are missing. The policy recognizes adaptation but does not highlight CC mitigation measures and strategies. It is stated that adaption measures be worked out to mitigate impacts of CC. PB’s Water Policy (2018) highlights environmental hazards and its EPA recognizes the environment as a policy objective, aiming to “ensure effective enforcement of regulations for managing the health of acquirers in collaboration with EPA.” CC adaptation and mitigation mainstreaming are not detailed in any water sector policy.

#### Energy Sector Policies

Pakistan’s energy sector contributes 51% to the country’s total greenhouse gas emissions (Ashfaq 2017). Federal and provincial governments’ energy policy documents mention the need for environmental protection but fail to consider CC adaptation and mitigation. The Federal Power Policy (2013) focuses on energy affordability, efficiency, financial viability (GoP 2013b), and the need of a green building code. However, it too fails to mention CC. The same is the case for the Power Generation Policy 2015, which does recognize environmental safeguards as one of the policy objectives, though (GoP 2015b).

None of the provincial policies consider CC. The PB Power Generation Policy includes environmental protection as a policy objective and states that all the “requirements as related to EIA and NEQS will be met” (GoPB 2009). Furthermore, the KP Hydropower Policy 2016 recognizes that “requirements laid down by KP EPA and rules and regulations thereunder relating to NEQS and EIA shall have to be met” (GoKP 2016a, b), but fails to address inter-provisional environmental problems. The Government of GB has not formulated their own policy for power generation. Rather, it has adopted the Federal Power Generation Policy 2015.

#### Agriculture Sector Policies

The agriculture sector in Pakistan accounts for 43% of total national greenhouse gas emissions (GoPB 2018b). The National Food Security Policy states that the sector needs to “flexibly adapt into CC and be resilient enough to quickly recover from shocks and emergencies” (GoP 2018a, p. 25). Furthermore, whilst it recognizes policy measures for environmental biodiversity conservation and development of climate smart crops, it fails to highlight CC. Whilst CC adaptation measures are mentioned in PB’s Agriculture Policy 2018, KP’s Agriculture Policy mentions both, CC adaptation and mitigation measures and strategies for water, agriculture, and energy sectors (GoKP 2013). The GB Agriculture Sector Policy finally is quiet about CC (GoGB 2018b).

#### CC Policies

Pakistan’s first NCCP 2012 recognized the need to integrate CC adaptation and mitigation measures into sector planning, including water, agriculture, energy, transport, forestry, vulnerable ecosystems, and industrial sectors. It called for a development of plans at federal and provincial levels for effective NCCP implementation (GoP 2012b). The policy aims at integrating CCP with other inter-related national policies “to ensure water security, food security, and energy security of the country in the face of the challenges posed by CC” (GoP 2012b). The NCCP implementation framework (2014–2030) provides a mechanism to mainstream CC concerns into national planning to promote climate-compatible development at federal and provincial levels (GoP 2013a). However, neither NCCP 2012, nor its implementation framework identify a mechanism to evaluate CC adaptation and mitigation progress. Similarly, provisional governments have no approved CCPs yet, while draft CCPs are failing to consider PC with policies of water, agriculture, and energy sectors. PB’s CC policy draft (2017) recognized the need to be in line with the NCCP (2012), its implementation framework (2013) and PB’s Growth Strategy (2018) (GoPB 2018c). The policy objective is to “integrate climate-compatible development paradigm through climate resilient, low carbon, and water–energy–food nexus-related measures into key relevant sectors policies, strategies, and plan” (GoPB 2017). However, it fails to mention coordination mechanisms with cross sector policies. Similarly, KP Province’s CCP (2016) aims to “Integrate adaptation and mitigation measures into key relevant sectors policies, strategies, and plans” (GoKP 2016a; b) to “ensure water, food and energy security for KP province in the face of a CC”. In addition to CC adaptation for agriculture, water resources, forestry, and disaster preparedness, the KP CCP also recognizes CC mitigation measures for energy, transport, waste, industries, and urban planning (GoKP 2016a, b). The need to work in line with NCCP is acknowledged, but no coordination mechanisms with sector policies are mentioned. Similarly, the GB CC Strategy and Action Plan (2017) recognize the hydropower potential of the region, and CC impacts on glaciers, agriculture, and energy sectors (GoGB 2017b). Policy objectives, strategies, and actions to adopt and mitigate CC are acknowledged, but no implementation coordination mechanisms with other sectors are mentioned.

#### Development Plans and Strategies

Federal and PB’s development plans consider CC adaptation and mitigation measures, and introduce projects to address CC along with environmental protection. Similarly, the development plans of GB (GoGB 2017a) and KP (GoKP 2018a) recognize some schemes and measures for flood and environmental management but fail to integrate CC. The National Sustainable Development Strategy takes into consideration the three pillars of sustainable development: economic, social and environmental, and recognizes 17 SDGs with strategic objectives and targets, and commits to integrating CC and the environment into national and provincial sectoral polices, plans, and strategies (GoP 2017b).

PB’s Growth Strategy 2023 integrates the environment and CC and recognizes SDG-13 (Climate Action), which is about “[taking] urgent action to combat CC and its impacts” and SDG-12 “ensuring sustainable consumption and production patterns” structural and non-structural adaptation and mitigation measures, but lacks an implementation framework (GoPB 2019). Similarly, the KP development strategy considers CC adaptation and mitigation measures, environmental security, and the mainstreaming of environmentally friendly strategies to reduce environmental hazards (GoKP 2014; 2019b).

The sustainable development strategy of GB recognizes flood disasters and aims at ensuring the environment is considered in planning. Furthermore, it commits to conducting SEA in the water and energy sectors, but fails to explicitly consider CC (GoP and IUCN 2003).

#### Disaster [Risk] Management Plans (DMPs)

The Federal Government’s DMP 2012–2022 (GoP 2012c), PB’s DMP 2008 (GoPB 2008), and GB’s DMP 2008 (GoGB 2008) consider CC and the environment, and recognize structural and non-structural adaptation and mitigation measures for CC, DRR, and environmental protection. KP has not developed a DMP yet. However, there are monsoon contingency plans (GoKP 2017; 2018b; 2019a), focusing on flood disasters. Environment, CC structural and non-structural adaptation, and mitigation measures and DRR strategies are mentioned for vulnerable sectors (not including the energy sector).

### PC across Policies, Plans and Strategies at Federal and Provincial Levels

PC is not explicitly addressed in most policy documents. The Federal water policy considers NEP 2005 and NCCP 2012, but lacks coherence with agriculture and energy sector policies (GoP 2018b). Similarly, PB’s water policy highlights the need to adopt the national water policy measures and NCCP, but fails to establish working coordination with energy sector policies. Likewise, the KP drinking water policy highlights the need to adopt national drinking water policy and to align this with NEP 2005, but fails to mention policy coordination with CC, agriculture, and energy sector policies.

The Federal national food security policy states that “there is a need to implement NEP 2005 and NCCP 2012 in letter and spirit” (GoP 2018a, p.17, 19), but fails to consider policy coordination with energy sector policies. Likewise, the agriculture policies of PB and KP fail to state coordination with sectoral policies of water, energy, and CC policy. Also, the KP agriculture policy does not mention environmental policy. Similarly, although the agriculture sector policy of GB establishes a need to harmonize sectoral policies, it fails to mention water and energy, environmental, CC policies, and coherence mechanisms.

The Federal and PB’s power sector policies fail to mention coordination with policies of water, agriculture, CC, and environmental sectors. PB’s CC policy recognizes the NCCP and national energy policy, and is committed to a centralized provincial water policy. It also asks for PB’s power generation policy to be updated (GoPB 2017). However, it fails to consider coordination with agriculture policy. Similarly, the KP CC policy highlights the need to work in line with NCCP (GoKP 2016a, b), but fails to consider coordination with water, agriculture, and energy policies. The GB CC strategy recognizes the need to develop an energy policy, highlights NEP, and follows NCCP guidelines (GoGB 2017b). However, sectoral policies of water, agriculture, and energy are not considered. Similarly, federal and provincial governments’ development plans fail to consider environmental and CC policies.

PB’s Growth Strategy (GoPB 2019) does not consider CC policy while the sustainable development strategy of KP province fails to mention agriculture policy (GoKP 2019b). Similarly, the GB strategy for sustainable development 2003 fails to mention CC policy. Furthermore, there is no coordination with water, agriculture, and energy sector policies. The DMP of federal and provincial governments, of PB, GB, and KP fail to mention environmental, water, food and agriculture, as well as energy sectors policies.

### PC across CPEC and Sectoral Policies, Development Plans, and Strategies

The federal and provincial governments’ agriculture policies and development strategies consider the CPEC plan as an opportunity. For example, PB’s agriculture policy recognizes that CPEC connectivity enhances competitiveness with global and domestic markets but fails to mention coordination and implementation mechanisms. Similarly, the national food security policy recognizes the establishment of agricultural economic zones along CPEC routes, enhancing agricultural economic cooperation under CPEC, but is missing a policy coordination mechanism.

The water and energy sector policies miss integration with the CPEC plan 2017–2030. Likewise, Federal and KP development plans recognize development projects under the CPEC portfolio. The CPEC plan recognizes opportunities and possible implementation challenges, highlighting that industrialization and urbanization in Pakistan will speed up, whilst also acknowledging associated environmental degradation (Alam et al. 2007) and additional CC emissions.

The CPEC plan 2017–2030, whilst neither mentioning CC policies, nor CC adaptation and mitigation measures, highlights the need to optimize sourcing and technology development of the coal industry, the promotion of river planning for hydropower, wind, solar-energy development, and water efficient technology for irrigation. Moreover, the plan also recognizes the need for social environment safeguards for CPEC projects.

For industrial cooperation, the CPEC plan highlights the need to promote environmentally friendly processes without referring to NEP 2005 and the national operational strategy 2006 for clean development mechanisms. Similarly, although it highlights measures in sectoral policies, it does not mention sectoral polices of water, agriculture, and energy, DRR and CC PC mechanisms.

**Fig. 2:**
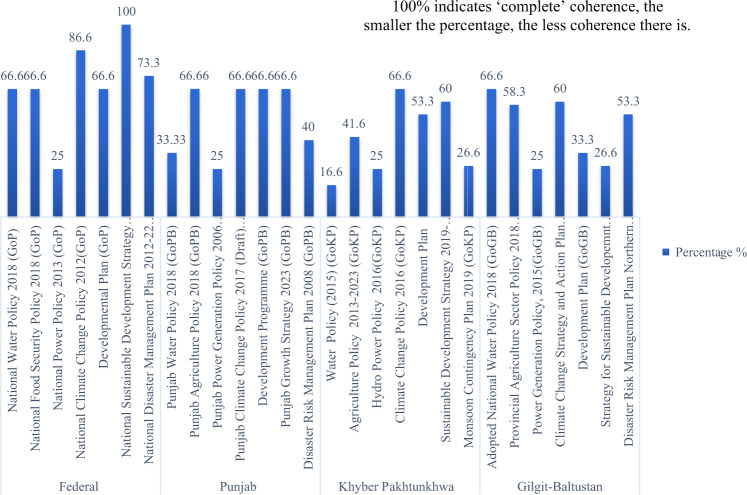
Coherence across policies, plans, and strategies for five key selected themes

**Fig. 3:**
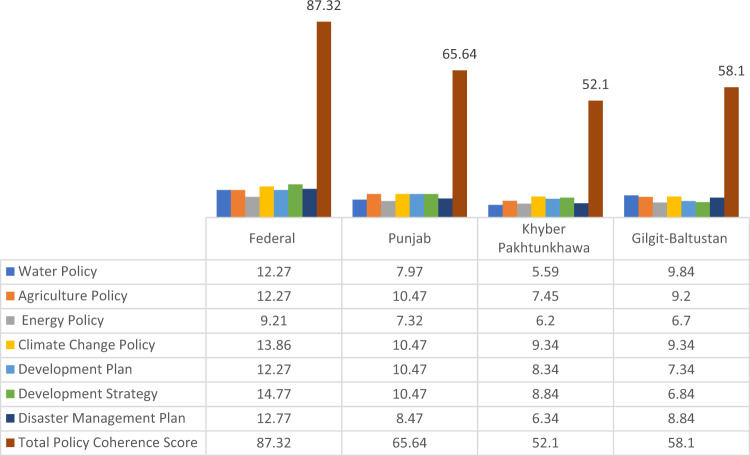
Overview of coherence score of policies, plans, and strategies of Fereral and Provincial governments

**Fig. 4:**
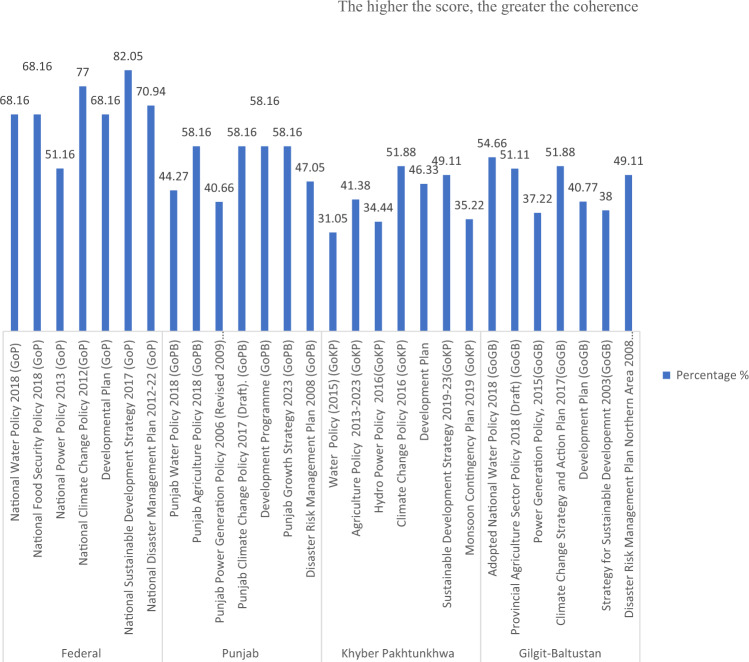
Coherence score across sectoral policies documents, strategies, and plans of Federal and Provincial level

Our analysis indicates that Federal, PB and GB water and agriculture policies were most coherent (Table 3). For example, the percent coherence score of the Federal Water Policy (2018) is 66.66% (see Table 3). The results of coherence analysis at federal and provincial levels across sectoral policies, CC policies, development strategies and plans, and disaster management plans are shown in Table 4 and Figs. 2–4.

With regards to PC on CC, the maximum possible coherence score would be 126. CC adaptation and mitigation mainstreaming in sectoral policies and other selected documents remains partial, with no scores of 3 for any Federal and Provincial policies being achieved. The analysis also indicates that energy sector policies at federal and provincial levels have the lowest PC scores to address CC.

Our analysis indicates that Federal water and agriculture sector policies were the most coherent documents, followed by PB, KP, and GB region. These coherence scores across policies are established by calculating the percent coherence value of each policy document (Table 4). For example, the percent coherence score of Federal’s Water Policy (2018) across policies is 68.16% (see Table 4).

## Discussion

CC (Janjua et al. 2018; Kouser et al. 2020) as well as environmental impacts (e.g., air and water quality) are the main challenges of Chinese FDI in Pakistan (Huang et al. 2017). Most energy projects of CPEC are coal-based (Faisal Mehmood et al. 2019). Northern Pakistan, a gateway to CPEC, has highly vulnerable and sensitive ecosystems (Dadwal and Purushothaman 2017) that are threatened, in particular by CPEC-funded highways (Nabi et al. 2018). Glaciers in Northern Pakistan cover an area of 5218 km^2^ (Gilany and Iqbal 2016). These glaciers are regulating local and global climate functions (Kääb et al. 2012). Glaciers in the region are currently receding rapidly (Gilany and Iqbal 2016) at a rate of 0.66 ± 0.09 m per year (Kääb et al. 2012). CPEC is likely to accelerate melting of these glaciers (Nabi et al. 2018).

Generally speaking, CPEC is expected to lead to a transition from rural to urban economies. Currently, water demands of urban lifestyles are much higher than those of rural settings, and CPEC projects are predicted to lead to an increased water demand for development that could exceed 2467 mm^3^ by 2025 and 4317 mm^3^ by 2050 (Amir and Habib 2015).

Pakistan’s energy sector contributes significantly to GHG emissions. It is projected that GHG emissions (in million tons CO_2_ eq.) will increase from 347 to 4621 in 2050 under a Business as Usual scenario (GoP and UNFCC 2011), and the energy sector will contribute almost 60% by the middle of the century (Khan et al. 2016). This is a challenge to CPEC development and could partly be addressed through CC mainstreaming in energy policies and PC, which is currently missing. The urbanization and industrialization agendas under the CPEC plan portfolio further enhance environmental degradation and CC impacts. CC PC is essential for synergies and sustainable development (Di Gregorio et al. 2017) and the CPEC plan fails to consider environmental sustainability and CC PC, mainly because it fails to highlight ways of mitigating CC and environmental impacts. There is a multidimensional interrelationship linkage among development, environmental degradation, and climate-driven disaster events (Gore and Fischer 2014). Therefore, challenges from the CPEC plan include CC and interlinked environmental degradation, as well as climate-driven disasters.

Whilst the CPEC plan recognizes the multidimensional aspects connected with the economic cooperation with China (and with other regional countries), it fails to establish PC mechanisms to deal with environmental and CC global issues. However, the country has legal instruments for implementation of environmental and CC policies, including the Federal and provincial environmental acts and Pakistan’s CC Act 2017. It means that the country is progressing with regards to its institutional and legal framework but lacks CC PC. In this context, SEA, a tool to promote CC PC and CC mainstreaming of policies, plans, and programs (Barker and Fischer 2003) is included in provincial environmental acts only. CC PC is challenging for Pakistan in particular when considering that currently the government is spending just 0.00028% of GDP on environmental protection, including CC mitigation and adaptation (Khan 2016).

The Federal Power Policy (2013) plans for coal-based energy production of 6000–7000 MW, and also mentions medium and long-term plans for hydro, solar, and wind projects, including 2726 MW of wind electricity, 341 MW of solar energy, and the possibility for hydropower projects of Bunji (7100 MW potential) and Diamer-Bhasha (4500 MW potential) (GoP 2013b). The Diamer-Bhasha dam is meant to also mitigate floods (APP 2020) and provide low-cost and clean electricity (Gul 2020). Federal government has set a target of 30% of renewable energy generation by 2030 (GoP 2019a, b), in line with an international obligation to reduce GHG emissions. The federal power policy 2013 failed to establish targets for renewable energy generation. Similarly, PB’s Power Policy and KP’s Hydropower Policy (2016) recognize the hydro potential and aim at generating hydropower of 600 MW at Federal (GoPB 2009) and 30,000 MW (GoKP 2016a, b) at provincial levels. Pakistan has a hydropower potential of 100,000 MW (Qazilbash 2015) and whilst the government recognizes this potential there is currently no coherent mechanism for implementation.

Pakistan has been portrayed as a country with low CC adaptive capacity (Abid et al. 2015). And whilst it is contributing only 0.8% of the total global GHG budget (Hussain et al. 2018), it has committed to reduce carbon emissions by 20% as part of ‘Intended Nationally Determined Contributions’ (GoP 2016). To address CC, Pakistan has CC policies at federal and provincial levels. The Federal NCCP (2012) highlights CC mitigation measures for sectors such as energy, forestry, agriculture, and livestock. The main CC mitigation targets focus on GHGs emissions reduction in energy and agriculture sectors, with special attention given to energy efficiency, while other highlighted mitigation areas include changes to the energy mix, renewable energy resource development, and increased share of nuclear and hydroelectric power. The country also recognizes that if it wants to tap into its coal resources of 185 billion tons, clean coal technologies will be needed (GoP 2012b). Green fiscal reforms in the energy and water sectors to reduce carbon emissions are also anticipated.

In the PB CC Policy 2017 a “Triple Win” strategy is advocated where the need for carbon compatible and climate resilient development is stressed and where co-benefits (water–energy–food nexus) are considered. The policy recognizes the need to implement CC adaptation and mitigation measures for NCCP projects. The policy highlights a 4000-MW demand–supply gap (GoPB 2017), which needs to be addressed through energy efficiency gains and by supporting renewable power plants. The policy also establishes that coal-based power plants need to adopt pollution control technologies.

The KP CCP highlights CC adaptation policy measures for agriculture and water resources. Similarly, it recognizes the hydropower potential of the province and highlights climate mitigation policy measures, for example indigenous renewable energy resources and zero emission strategies, use of solar water heating technologies, and financial incentives, including carbon taxes, subsidies, and tax reforms. Other measures include energy efficiency standards and measures, the promotion of energy efficiency technologies, and awareness raising for energy saving measures. The GB CC Strategy and Action Plan (2017) recognize the need to mainstream CC mitigation and adaptation, highlighting strategies, objectives and action plans for thematic areas. These include e.g., infrastructure resilience, CC mitigation and adaptation for water, and power sectors. It highlights that current energy demand of GB is more than 500 MW while the hydropower generation potential in GB is more than 40,000 MW (GoGB 2017b).

Pakistan is ranked 7th internationally regarding coal reserves (Khosa and Malkani 2016) with about 186 billion tons of coal (Ghaznavi et al. 2016) resources in all provinces (Malkani et al. 2016). Coal is currently the most significant and cost-effective energy source in Pakistan (Satti et al. 2014). Thar coal alone has the potential of 100,000-MW electricity generation (Tribune 2020). KP province and GB have about 76% of country’s total hydro generation potential of 45,861 MW, while PB and AK have a potential of 7291 and 6450 MW, respectively (Wajsal 2015). However, 89% of this potential still remains unexploited. In addition to the wind energy potential of more than 300,000 MW (Wajsal 2015), Pakistan has 2.9 million MW of solar-energy potential (Tribune 2017, Wajsal 2015). About 40,000 remote villages will receive solar-energy-based electricity (Khan et al. 2014). Solar energy is the best renewable energy option for Pakistan in terms of maintenance cost, operations, and life span (Irfan et al. 2019). The country receives solar radiation of 1500–2500 h annually (Kiani 2019) and Balochistan alone has an annual mean sunshine duration of 8–8.5 h per day (Kiani 2019), the highest solar potential in the world.

With regards to PC around CC adaptation and mitigation, Federal Government has the most coherent set of policy documents in place with a total coherence score of 87.32, followed by PB (65.64), GB (58.1), and then KP (51.1) (see Table 4). At Federal level, the National Sustainable development Strategy 2017 has the highest PC score (14.77), followed by NCCP 2012 (13.86) and DMP 2012–2022 (12.77). The National Water Policy 2018, and National Food Policy Security Policy 2018, and Development Plan have a PC score of 12.27. The Energy Policy has the lowest PC score (9.21).

It is important that in Pakistan, entire policy documents are simply copied. For example, GB has adopted federal water and energy sector policies. The problem with simply using federal policy is that they were not developed in consultation with the relevant stakeholders. Such adopted policies are bound to fail to establish coherence with other sector policies. Dependency on policies developed by external stakeholders reduces opportunities for consultation across relevant departments and ministries, meaning that opportunities for greater coherence are being missed. Policies remaining in draft form for a long time have been found to reduce PC throughout the world. Policy evaluation and revision are key for effective PC development (Picciotto 2005). A common issue of developing countries is infrequent and delayed policy revisions, as well as missing designated specific review dates. Most of the sectoral policies have rarely been revised. For example, Tanzania’s national water policy was first developed in 1991 and revised in 2002, while national agriculture policy was first produced in 2013 (England et al. 2018). In case of Pakistan’s policy documents, they are also rarely updated and approved. Water sector policy formation and approval processes are very slow in Pakistan. The country’s first national water policy was approved as recently as 2018 (after many years of having draft status (Khalid 2017), while Malawi’s, Tanzania’s, and Zambia’s National Water Policies are from 2005, 2002, and 1994, respectively.

## Conclusions

Pakistan’s national sustainable development strategy (2017) recognizes the necessity to enhance PC as a strategic objective. However, this paper has shown that only some policies, strategies, and plans are being coordinated with other sectoral policies and plans. An effective mechanism of maintaining coherence is missing in Pakistan. CC adaptation and mitigation is covered in all water and agriculture sector policies but not in energy sector policies. This has implications for the CPEC Plan (2017–2030), which has remained a standalone document as it does not consider the country’s CC policies, sectoral policies, and PCPC mechanisms. When a mega development plan of power generation and infrastructure development projects with expected significant environmental impacts is being executed under the CPEC, there should be coherence with CC, water, agriculture and energy sector policies and plans. Provincial draft CC policies, and the GB regional strategy for sustainable development 2003 need revisions with a PC approach in mind. After the 18th constitutional amendment, environment and disaster management have become provincial matters. Therefore, NEP 2005 and Provincial DRR plans of 2008 and of GB region need to be updated and revised. Janetschek et al. (2020) suggested that active engagement of stakeholders is essential for effective PC for development and mutual benefits in national policies. This is currently missing in Pakistan (Ghani 2014; Husain 2013). There is a need to conduct effective stakeholder consultation in policy formation and for existing sectoral policies and CPEC plan revisions to ensure CC mainstreaming and PC for development. It is recommended that CC and environmental concerns need to be integrated into energy sector policies as most of the power generation projects under CPEC are coal-based and are likely to contribute significantly to greenhouse gas emissions. The government should establish an inter-ministerial committee or a similar body for devising mechanisms at federal and provincial levels for PC to address CC and environmental impacts. In general terms, in Pakistan the environment and CC remain subject to a supply rather than a demand-driven agenda. The CPEC act as a bridge for BRI development. In January 2021, 140 countries of Asia, Europe, Africa, and beyond had joined the BRI initiative (Nedopil 2021). CC and environmental degradation are substantial challenges of BRI development (Desheng et al. 2018; Fang et al. 2021; Deng and Du 2020). CC PC has important implications for countries along the BRI with regards carbon reduction targets, and with regards to achieving coordinated socio-economic and ecological development.

## References

[CR1] Abid M, Scheffran J, Schneider UA, Ashfaq M (2015). Farmers’ perceptions of and adaptation strategies to climate change and their determinants: the case of Punjab province, Pakistan. Earth Syst Dyn.

[CR2] Ahmed T, Zounemat-Kermani M, Scholz M (2020). Climate change, water quality and water-related challenges: a review with focus on Pakistan.. Int J Environ Res Public Health.

[CR3] Alam S, Fatima A, Butt MS (2007). Sustainable development in Pakistan in the context of energy consumption demand and environmental degradation. J Asian Econ.

[CR4] Ali R, Kandhro SH (2015). National Disaster Management Authority in Pakistan: role of Pakistan army in disaster management. J Soc Adm Sci.

[CR5] Altheide D, Coyle M, Devriese K, Schneider C (2008) Emergent qualitative document analysis. In: HESSE-BIBER, S. N. &LEAVY, P. (eds.) Handbook of emergent methods. The Guilford Press, New York, NY, p 127–151

[CR6] Amir P, Habib Z (2015) Act report estimating the impacts of climate change on sectoral water demand in Pakistan. Action on Climate today

[CR7] APP (2020) Government acquires 86% land for Diamer-Bhasha dam. Associated Press of Pakistan (APP) https://www.app.com.pk/national/86-land-acquired-for-diamer-basha-dam-so-far/. 01 Nov 2020

[CR8] Ashfaq Z (2017) Impact of 18 constitutional amendment on governance of renewable energy in Pakistan. WWEA Policy Paper Series (PP-02-17), World Wind Energy Associaon (WWEA)

[CR10] Bank W (2006) Pakistan Strategic Country Environmental Assessment. Main report. Report

[CR11] Barker A, Fischer TB (2003). English regionalization and sustainability: towards the development of an integrated approach to strategic environmental assessment. Eur Plan Stud.

[CR12] Benson D, Lorenzoni I (2017). Climate change adaptation, flood risks and policy coherence in integrated water resources management in England. Regional Environ Change.

[CR13] Bilgen S (2016). The environmental effects of coal-related activities. Energy Sources, Part A: Recover, Util, Environ Eff.

[CR14] Birkmann J, Von Teichman K (2010). Integrating disaster risk reduction and climate change adaptation: key challenges—scales, knowledge, and norms. Sustain Sci.

[CR15] Chaudhry QUZ (2017) Climate change profile of Pakistan. Asian Development Bank, Philippines

[CR16] Cohen PJ, Song AM, Morrison TH (2017) Policy coherence with the small-scale fisheries guidelines: analysing across scales of governance in Pacific small-scale fisheries. In: The Small-scale fisheries guidelines. Springer International Publishing, Cham

[CR17] Dadwal SR, Purushothaman C (2017). CPEC in Pakistan’s quest for energy security. Strategic Anal.

[CR18] Deng X, Du L (2020). Estimating the environmental efficiency, productivity, and shadow price of carbon dioxide emissions for the Belt and Road Initiative countries.. J Clean Prod.

[CR19] Desheng H, Jun O, Xueyue H (2018) On the environmental responsibility of chinese enterprises for their FDIs in countries within the belt and road initiative. Springer International Publishing, Cham

[CR20] Di Gregorio M, Nurrochmat DR, Paavola J, Sari IM, Fatorelli L, Pramova E, Locatelli B, Brockhaus M, Kusumadewi SD (2017). Climate policy integration in the land use sector: mitigation, adaptation and sustainable development linkages. Environ Sci Policy.

[CR21] Ebrahim ZT (2020) How CPEC projects will increase Pakistan’s reliance on coal – and emissions [Online]. Science THE Wire. https://science.thewire.in/environment/cpec-china-pakistan-military-environment-coal/. Accessed 10 Oct 2020

[CR22] Eckstein D, Hutfils M-L, Winges M (2018) Global Climate Risk Index 2019: who suffers most from extreme weather events? Weather-related loss events in 2017 and 1998 to 2017. Germanwatch Nord-Süd Initiative EV, Berlin

[CR23] England MI, Dougill AJ, Stringer LC, Vincent KE, Pardoe J, Kalaba FK, Mkwambisi DD, Namaganda E, Afionis S (2018). Climate change adaptation and cross-sectoral policy coherence in southern Africa. Reg Environ Change.

[CR24] Faisal Mehmood M, Nishat F, Kafait U (2019). Impact of China-Pakistan economic corridor on Pakistan’s future energy consumption and energy saving potential: evidence from sectoral time series analysis. Energy Strategy Rev.

[CR25] Fang K, Wang S, He J, Song J, Fang C, Jia X (2021). Mapping the environmental footprints of nations partnering the Belt and Road Initiative. Resour, Conserv Recycl.

[CR26] Farooqui MA, Aftab SM (2018) China-Pakistan economic corridor; prospects and challenges for Balochistan, Pakistan. Proceedings of the IOP Conference Series: Materials Science and Engineering, p. 012046

[CR27] Fischer TB (2007) The theory and practice of strategic environmental assessment: towards a more systematic approach. Earthscan, London Sterling

[CR28] Gerston L (2010) Public policy making: process and principles. Routledge, London

[CR29] Ghani A (2014) In Pakistan, policy-making is largely done by bureaucracy. The News on Sunday (TNS). https://www.thenews.com.pk/tns/detail/554602-pakistan-policy-making-largely-done-bureaucracy

[CR30] Ghani WI, Sharma R (2018). China-Pakistan Economic Corridor Agreement: impact on shareholders of Pakistani Firms. Int J Econ Financ.

[CR31] Ghaznavi MI, Bukhari SMR, Altaf KH (2016). Coal resources of Pakistan with special references to some coalfields of KPK. J Himal Earth Sci.

[CR32] Gilany SNA, Iqbal J (2016). Geospatial analysis of glacial hazard prone areas of Shigar and Shayok basins. Int J Innov Appl Stud.

[CR33] GoGB (2008) Disaster Risk Mangement Plan Northern Areas, Government of Gilgit-Baltustan,. In: Authority N. A. D. M. (ed) http://web.ndma.gov.pk/plans/Provincial%20DRM%20Plan%20NorthernAreas.pdf. 20 Dec 2019

[CR34] GoGB (2017a). Annual Development Program. Government of Gilgit-Baltistan. In: Department P. A. D. (ed) http://www.gilgitbaltistan.gov.pk/DownloadFiles/ADPS/ADP201718.pdf. 5 Jan 2020

[CR35] GoGB (2017b) Gilgit-Baltistan climate change strategy and action plan. Government of Gilgit-Baltustan. In: Agency G.-B. E. P. (ed) http://gbepa.gog.pk/. 10 Dec 2019

[CR36] GoGB (2018a) National Water Policy. Government of Pakistan. Resources M. O. W., Islamabad, http://mowr.gov.pk/wp-content/uploads/2018/06/National-Water-policy-2018-2.pdf. 2 July 2019

[CR37] GoGB (2018b) Provincial Agriculture Sector Policy. Government of Gilgit-Balltistan. In: Department of Agriculture L. A. F (ed), https://www.govserv.org/PK/Gilgit/355726594874296/Agriculture%2C-Livestock-%26-Fisheries-Department-Gilgit-Baltistan. 2 Feb 2019.

[CR38] GoKP (2013) Agriculture Policy Khyber Pakhtunkhwa a ten year perspective (2013–2023). Government of Khyber Pakhtunkhwa. Government of Khyber Pakhtunkhwa. Department of Agriculture L. A. C. http://www.fao.org/3/a-at525e.pdf. 10 Nov 2019

[CR39] GoKP (2014) Integrated Development Strategy (2014–18). Government of Khyber Pakhtunkhwa (GoKP). PROGRAMME K. P. C.-D. L. D. C. http://cdldta.pk/downloads/publications/Khyber%20Pakhtunkhwa%20Integrated%20Development%20Strategy%202014-2018.pdf. 5 Oct 2019.

[CR40] GoKP (2015) Drinking Water Policy. Government of Khyber Pakhtunkhwa. Public Health Engineering Department. https://phedkp.gov.pk/downloads.php. 11 Dec 2019

[CR41] GoKP (2016a) Khyber Pakthunkhwa Climte Change Policy. Government of Khyber Pakhtunkhwa. Environmental Protection Agency. FORESTRY, E. W. D. http://kp.gov.pk/uploads/2016/11/Final_Climate_Change_Policy_for_KP_Province_25_October,_2016_WebSec_Comments.pdf. 10 Oct 2019

[CR42] GoKP (2016b) Pakhtunkhwa Hydropower Policy. Government of Khyber Pakhtunkhawa. Organization P. E. D. http://www.kpkep.gov.pk/documents/KPK%20Hydel%20Power%20Policy%202016.pdf. 19 Aug 2019

[CR43] GoKP (2017) Monsoon Contingency Plan. Government of Khyber Pakhtunkhwa. Authority P. D. M. http://pdma.gov.pk/downloads.php. 20 Dec 2020

[CR44] GoKP (2018a) Annual Development Plan 2018–19. Government of Khyber Pakhtunkhwa (GoKP). Department P. A. D. https://pndkp.gov.pk/data/sogo/trace/adp/15947948505f0e97a0adcfe.pdf. 12 Nov 2019

[CR45] GoKP (2018b) Monsoon Contingency Plan. Government of Khyber Pakhtunkhwa. Authority P. D. M. http://pdma.gov.pk/downloads.php. 10 Jan 2020

[CR46] GoKP (2019a) Monsoon Contingency Plan. Government of Khyber Pakhtunkhwa (GoKP). Authority P. D. M., Peshawar, Pakistan. http://www.pdma.gov.pk/sites/default/files/Monsoon%20Contingency%20Plan%202019_0.pdf.dated. 25 Jan 2020

[CR47] GoKP (2019b) Sustainable Development Strategy a medium term development framework for Khyber Pakhtunkhwa. Government of Khyber Pakhtunkhwa (GoKP). Department P. A. D. https://pndkp.gov.pk/data/sogo/trace/downloads/15833297365e5f973890459.pdf. 22 Nov 2019

[CR48] GoP (2010) Task Force on Climate Change: final report. Commission P. HTTP://WWW.MOCC.GOV.PK/MOCLC/USERFILES1/FILE/MOC/PUBLICATIONS%20ON%20ENV%20AND%20CC/REPORTS/TFCC%20FINAL%20REPORT%2019%20FEB%202010.PDF. 17 Nov

[CR49] GoP (2012a) Annual flood report 2012. Government of Pakistan. Power M. O. W. A. Federal Flood Commission, Islamabad. http://mowr.gov.pk/wp-content/uploads/2018/06/Annual-Flood-Report-2012.pdf. 16 Dec 2019

[CR50] GoP (2012b) National Climate Change Policy. Government of Pakistan (GoP). Change M. O. C., Islamabad. http://www.mocc.gov.pk/frmDetails.aspx. 17 Sep 2019

[CR51] GoP (2012c) National Disaster Management Plan. Government of Pakistan (GoP). Change. M. O. C., National Disaster Management Authority. http://web.ndma.gov.pk/plans/NDMP-Main%20Vol.pdf. 20 Dec 2019

[CR52] GoP (2013a) Framework for Implementation of Climate Change Policy (2014–2030) Government of Pakistan(GoP). Change M. O. C., Islamabad, Pakistan. http://www.gcisc.org.pk/Framework%20for%20Implementation%20of%20CC%20Policy.pdf. 10 June 2019

[CR53] GoP 2013b. National Power Policy. Government of Pakistan (GoP). Power M. O. W. A., Islamanad. http://www.ppib.gov.pk/National%20Power%20Policy%202013.pdf. 7 Sep 2019

[CR54] GoP (2014) Pakistan 2025 one nation-one vsion. Government of Pakistan (GoP). Planning Commission. Ministry of Planning D. R., Islamabad. https://www.pc.gov.pk/uploads/vision2025/Pakistan-Vision-2025.pdf. 17 Sep 2019

[CR55] GoP (2015a) Pakistan Economic Survey 2014–15. Government of Pakistan (GoP). Division M. O. F., Islamabad. http://finance.gov.pk/survey/chapters_15/Highlights.pdf. 2 Oct 2019

[CR56] GoP (2015b) Power Generation Policy. Government of Pakistan (GoP). Power Mowa, Islamabad. http://www.ppib.gov.pk/Power%20Generation%20Policy%202015%20small.pdf. Aug 2019

[CR57] GoP (2016) Pakistan’s Intended Nationally Determined Contribution (Pak-INDC). Governemnt of Pakistan (GoP). Change M. O. C., Islamabad. https://www4.unfccc.int/sites/submissions/INDC/Published%20Documents/Pakistan/1/Pak-INDC.pdf. 17 Aug 2019

[CR58] GoP (2017a) Long term plan for for China-Pakistan economic corridor (2017–2030). Government of Pakistan (GoP) Planning Commission. Ministry of Planning D. R., Islamabad. http://cpec.gov.pk/long-term-plan-cpec. 10 Jun 2019

[CR59] GoP (2017b) National Sustainable Development Strategy. Government of Pakistan. Change M. O. C., Islamabad. http://www.mocc.gov.pk/

[CR60] GoP (2018a) National Food Security Policy. Government of Pakistan (GoP). Research M. O. N. F. S. A., Islamabad. http://mnfsr.gov.pk/userfiles1/file/National%20Food%20Security%20Policy%20%202018%20(1).pdf. 12 June 2019

[CR61] GoP (2018b) National Water Policy. Government of Pakistan. Ministry of Water Resources. Resources M. O. W., Islamabad. http://mowr.gov.pk/wp-content/uploads/2018/06/National-Water-policy-2018-2.pdf. 2 Jul 2019

[CR62] GoP (2019a) Annual Plan 2019–20. Government of Pakistan. Planning Commission. Ministry of Planning D. R., Islamabad. https://www.pc.gov.pk/uploads/annualplan/AnnualPlan2019-20.pdf. 19 Dec 2019

[CR63] GoP (2019b) Power plicy alternative and revewable energy. Government of Pakistan. In: DIVISION, M. O. E. P. (ed.). Alternative Energy Development Board Islamabad. http://www.aedb.org/images/ARE_Policy_2019_-_Gazette_Notified.pdf. 2 Nov 2020

[CR64] GoP, IUCN (2003) Northern areas strategy for sustainable development. Government of Pakistan and International Union for Conservation of Nature (IUCN), PAKISTAN, K. X. P., Pakistan. https://portals.iucn.org/library/sites/library/files/documents/2003-095.pdf. 6 Nov 2019

[CR65] GoP, UNFCC (2011) National Economic & Environmental Development Study Pakistan. Government of Pakistan (GOP) and United Nations Framework Convention on Climate Change (UNFCCC). Pakistan M. O. E., Islamabad. https://unfccc.int/sites/default/files/pakistanneeds.pdf. 15 Jan 2020

[CR66] GoPB (2008) Disaster Risk Management Plan Punjab. Governemnt of Punjab (GoPB). Authority P. D. M., Punjab. http://web.ndma.gov.pk/plans/Provincial%20DRM%20Plan%20Punjab.pdf. 10 Nov 2019

[CR67] GoPB (2009) Punjab Power Generation Policy, 2006 (Revised 2009) Governemnt of Punjab (GoPB). Board, P. P. D. https://energy.punjab.gov.pk/governing_laws_and_policies. 14 Nov 2019

[CR69] GoPB (2018a) Delopment Programme 2018–19. Midium Term Development Framwork 2018–21. Government of Punjab (GoPB). Board P. A. D. https://pnd.punjab.gov.pk/. 20 Dec 2019

[CR68] GoPB (2017) Punjab Climate Change Policy draft. Government of Punjab (GoPB). In: DEPARTMENT., E. P. (ed.). Environment Protection Department Punjab. https://epd.punjab.gov.pk/system/files/PCCP%20Draft%20%28internatl%29_0.pdf. 15 Sep 2019

[CR70] GoPB (2018b) Punjab Agriculture Policy. Government of Punjab (GoPB). Department P. A. http://agripunjab.gov.pk/system/files/Punjab%20Agriculture%20Policy.pdf. 8 Sep 2019

[CR71] GoPB (2018c) Punjab Growth Strategy. Government of Punjab. Department P. D. https://pnd.punjab.gov.pk/system/files/Punjab_Growth_Strategy_2018_0.pdf. 6 Jan 2020

[CR72] GoPB (2018d) Punjab Water Policy. Government of Punjab. Department I. http://extwprlegs1.fao.org/docs/pdf/pak191275.pdf. 05 Dec 2019

[CR73] GoPB (2019) Punjab Growth Strategy. Government of Punjab (GoPB). Board PAD. https://pnd.punjab.gov.pk/system/files/PGS_2023%2019-21-145.pdf#overlay-context=economic_growth_strategy. 4 Feb 2020

[CR74] Gore T, Fischer TB (2014). Uncovering the factors that can support and impede post-disaster EIA practice in developing countries: the case of Aceh Province, Indonesia. Environ Impact Assess Rev.

[CR75] Gul A (2020) China’s Investments in Pakistan-Administered Kashmir Seen as ‘Blow’ to India. VOA News on China. https://www.voanews.com/east-asia-pacific/voa-news-china/chinas-investments-pakistan-administered-kashmir-seen-blow-india. 01 Nov 2020

[CR76] Hellin J, Beuchelt T, Camacho C, Govaerts B, Donnet L, Riis-Jacobsen J (2014) An innovation systems approach to enhanced farmer adoption of climate-ready germplasm and agronomic practices. International Food Policy Research Institute

[CR77] Howden SM, Soussana J-F, Tubiello FN, Chhetri N, Dunlop M, Meinke H (2007). Adapting agriculture to climate change.. Proc Natl Acad Sci USA.

[CR78] Howlett M, Rayner J (2007). Design principles for policy mixes: cohesion and coherence in ‘new governance arrangements’. Policy Soc.

[CR79] Huang Y, Fischer TB, Xu H (2017). The stakeholder analysis for SEA of Chinese foreign direct investment: the case of ‘One Belt, One Road’initiative in Pakistan. Impact Assess Proj Appraisal.

[CR80] Husain I (2013) Formulating public policy. Dawn. https://www.dawn.com/news/1056309

[CR81] Husain I, Arrfat Y (2018) CPEC and Pakistani economy: an appraisal. CPEC Center of Excellence, Pakistan ministry of Planning

[CR82] Hussain M, Butt AR, Uzma F, Ahmed R, Irshad S, Rehman A, Yousaf B (2019). A comprehensive review of climate change impacts, adaptation, and mitigation on environmental and natural calamities in Pakistan. Environ Monit Assess.

[CR83] Hussain M, Butt AR, Uzma F, Ahmed R, Islam T, Yousaf B (2019). A comprehensive review of sectorial contribution towards greenhouse gas emissions and progress in carbon capture and storage in Pakistan. Greenh Gases: Sci Technol.

[CR84] Hussain M, Liu G, Yousaf B, Ahmed R, Uzma F, Ali MU, Ullah H, Butt AR (2018). Regional and sectoral assessment on climate-change in Pakistan: social norms and indigenous perceptions on climate-change adaptation and mitigation in relation to global context.. JOCP.

[CR85] Irfan M, Zhao Z-Y, Ahmad M, Mukeshimana MC (2019). Solar energy development in Pakistan: Barriers and policy recommendations.. Sustainability.

[CR86] Janetschek H, Brandi C, Dzebo A, Hackmann B (2020). The 2030 Agenda and the Paris Agreement: voluntary contributions towards thematic policy coherence. Clim Policy.

[CR87] Janjua S, Khan A, Asif N (2018) Impact of CPEC on climate change—policy recommendations, Policy Paper #11. Islamabad. https://cpec-centre.pk/wp-content/uploads/2018/10/11-policy-paper.pdf

[CR88] Jiricka-Purrer A, Czachs C, Formayer H, Wachter TF, Margelik E, Leitner M, Fischer TB (2018). Climate change adaptation and EIA in Austria and Germany-current consideration and potential future entry points.. Environ Impact Assess Rev.

[CR89] Juhola S, Westerhoff L (2011). Challenges of adaptation to climate change across multiple scales: a case study of network governance in two European countries. Environ Sci Policy.

[CR90] Kääb A, Berthier E, Nuth C, Gardelle J, Arnaud Y (2012). Contrasting patterns of early twenty-first-century glacier mass change in the Himalayas. Nature.

[CR91] Kalaba F, Quinn C, Dougill A (2014). Policy coherence and interplay between Zambia’s forest, energy, agricultural and climate change policies and multilateral environmental agreements. Int Environ Agreem: Politics, Law Econ.

[CR92] Khalid D (2017) Pakistan’s National Water Policy. The Express Tribune. https://tribune.com.pk/story/1469030/pakistans-national-water-policy. 10 Feb 2020

[CR93] Khan AN, Jan MA (2015) National strategy, law and institutional framework for disaster risk reduction in Pakistan. In: Disaster risk reduction approaches in Pakistan. Springer

[CR94] Khan I (2016) Environmental degradation costs Pakistan Rs1 billion a day. Today’s Paper. https://www.thenews.com.pk/print/119575-Environmental-degradation-costs-Pakistan-Rs1-billion-a-day. 13 May 2016

[CR95] Khan MA, Khan JA, Ali Z, Ahmad I, Ahmad MN (2016). The challenge of climate change and policy response in Pakistan. Environ Earth Sci.

[CR96] Khan N, Mirza IA, Khalil MJ (2014) Renewable energy in Pakistan: status and trends. AEDB

[CR97] Khan S (2019). Policy diagnostics for Pakistan’s water security challenge. World Water Policy.

[CR98] Khosa MH, Malkani MS (2016). Coal resources of Sindh; discovery of large lignitic coal deposits in Thar. J Himal Earth Sci.

[CR99] Kiani DA (2019) Solar—the untapped potential in Pakistan. The Express Tribune

[CR100] Kouser S, Subhan A, Abedullah (2020). Uncovering Pakistan’s environmental risks and remedies under the China-Pakistan economic corridor.. Environ Sci Pollut Res.

[CR101] Le Gouais A, Wach E (2013). A qualitative analysis of rural water sector policy documents. Water Altern.

[CR102] Malik SM, Awan H, Khan N (2012). Mapping vulnerability to climate change and its repercussions on human health in Pakistan. Glob Health.

[CR103] Malkani MS, Alyani MI, Khosa MH, Buzdar FS (2016) Coal Resources of Pakistan: new coalfields. 5:7–22

[CR104] Mallory TG (2016). Fisheries subsidies in China: quantitative and qualitative assessment of policy coherence and effectiveness. Mar Policy.

[CR105] Mehar A (2017). Infrastructure development, CPEC and FDI in Pakistan: is there any connection?. Transnatl corporations Rev.

[CR106] Mirza FM, Fatima N, Ullah KJESR (2019). Impact of China-Pakistan economic corridor on Pakistan’s future energy consumption and energy saving potential: evidence from sectoral time series analysis. Energy Strategy Rev.

[CR107] Mohydin R (2019) Climate change does not need visas to cross borders. https://www.amnesty.org/en/latest/news/2019/06/pakistan-climate-change-does-not-need-visas-to-cross-borders/. Accessed 13 May 2020

[CR108] Mukhtar R (2018). Review of national multi-hazard early warning system plan of Pakistan in context with sendai framework for disaster risk reduction. Procedia Eng.

[CR109] Nabi G, Ullah S, Khan S, Ahmad S, Kumar S (2018). China-Pakistan Economic Corridor (CPEC): melting glaciers—a potential threat to ecosystem and biodiversity. Environ Sci Pollut Res.

[CR110] Nedopil C (2021) Countries of the belt and road initiative [Online]. Beijing International Institute of Green Finance Green BRI Center. www.green-bri.org. Accessed 5 Feb 2021

[CR111] Nilsson M, Zamparutti T, Petersen JE, Nykvist B, Rudberg P, Mcguinn J (2012). Understanding policy coherence: analytical framework and examples of sector–environment policy interactions in the EU. Environ Policy Gov.

[CR112] Papadopoulou C-A, Papadopoulou MP, Laspidou C, Munaretto S, Brouwer F (2020). Towards a low-carbon economy: a nexus-oriented policy coherence analysis in Greece.. Sustainability.

[CR113] Picciotto R (2005). The evaluation of policy coherence for development. Evaluation.

[CR114] Qazilbash IA (2015) Pakistan has 100,000MW production potential. The Express Tribune. https://tribune.com.pk/story/996382/hydroelectric-power-pakistan-has-100000mw-production-potentia. 23 Nov 2015

[CR115] Qazilbash Z (2017) CPEC—moving from discussion to solutions. The Express Tribune

[CR116] Qureshi A (2005). Climate change and water resources management in Pakistan. Climate change and water resources in South Asia..

[CR9] Rahman A-U (2010) Disaster risk management: Flood perspective. VDM Verlag Publishing Co. Ltd, Germany

[CR117] Ranabhat S, Ghate R, Bhatta LD, Agrawal NK, Tankha S (2018). Policy coherence and interplay between climate change adaptation policies and the forestry sector in Nepal. EM.

[CR118] Satti SL, Hassan MS, Mahmood H, Shahbaz M (2014). Coal consumption: an alternate energy resource to fuel economic growth in Pakistan. Econ Model.

[CR119] Scobie M (2016). Policy coherence in climate governance in Caribbean Small Island Developing States. Environ Sci Policy.

[CR120] Shaw R (2015) Introduction and approaches of disaster risk reduction in Pakistan. In: Disaster risk reduction approaches in Pakistan. Springer

[CR121] Siitonen L (2016) Theorising politics behind policy coherence for development (PCD). Springer

[CR122] Sirajul H (2015). Public policy process in Pakistan: key causes of public policies failures. J Econ Soc Thought.

[CR123] Steve S (2001). An overview of content analysis. Practical Assess, Res Eval.

[CR124] Tribune E (2017) Pakistan has ‘2.9 million MW solar energy potential’. The Express Tribune. https://tribune.com.pk/story/1357453/going-green-pakistan-1-9mw-solar-energy-potential. 17 Mar 2017

[CR125] Tribune E (2020) Coal use in Pakistan’s energy mix negligible. The Express Tribune. https://tribune.com.pk/story/2243411/2-coal-use-pakistans-energy-mix-negligible. 16 Jun 2020

[CR126] UN (2015) Sendai framework for disaster risk reduction 2015–2030. A. A. & 2019, W. U. O. F. S. P. D. O.

[CR127] UN (2016) General assembly takes action on second committee reports by adopting 37 texts. [Online]. United Nations. https://www.un.org/press/en/2016/ga11880.doc.htm

[CR128] UNFCC (2015) Adoption of the Paris agreement. UN, D. C. & Framework Convention on Climate Change. https://unfccc.int/resource/docs/2015/cop21/eng/10a01.pdf

[CR129] Vats R (2016) China-Pakistan economic corridor: energy and power play. Research Intern, Institute of Chinese Studies, New Delhi

[CR130] Verma M, Loha C, Sinha AN, Chatterjee PK (2017). Drying of biomass for utilising in co-firing with coal and its impact on environment—a review. Renew Sustain Energy Rev.

[CR131] Voyer M, Farmery AK, Kajlich L, Vachette A, Quirk G (2020). Assessing policy coherence and coordination in the sustainable development of a Blue Economy. A case study from Timor Leste.. Ocean Coast Manage.

[CR132] Wajsal (2015) Potential of renewable energies in Pakistan [Online]. Pakistan Defence. https://defence.pk/pdf/threads/potential-of-renewable-energies-in-pakistan.379477/. Accessed 11 Nov 2020

[CR133] Zubedi A, Jianqiu Z, Arain QA, Memon I, Khan S, Khan MS, Zhang Y (2018). Sustaining Low-Carbon Emission Development:An Energy Efficient Transportation Plan for CPEC.. J Inf Process Syst.

